# Dynamic causal modelling of distributed electromagnetic responses

**DOI:** 10.1016/j.neuroimage.2009.04.062

**Published:** 2009-08-15

**Authors:** Jean Daunizeau, Stefan J. Kiebel, Karl J. Friston

**Affiliations:** The Wellcome Trust Centre for Neuroimaging, Institute of Neurology, UCL 12 Queen Square, London, WC1N 3BG UK

**Keywords:** Dynamic causal modelling, EEG, MEG, Neural-mass, Neural-field, Variational Bayes, Inversion, System identification, Source reconstruction

## Abstract

In this note, we describe a variant of dynamic causal modelling for evoked responses as measured with electroencephalography or magnetoencephalography (EEG and MEG). We depart from equivalent current dipole formulations of DCM, and extend it to provide spatiotemporal source estimates that are spatially distributed. The spatial model is based upon *neural-field* equations that model neuronal activity on the cortical manifold. We approximate this description of electrocortical activity with a set of local standing-waves that are coupled though their temporal dynamics. The ensuing distributed DCM models source as a mixture of overlapping patches on the cortical mesh. Time-varying activity in this mixture, caused by activity in other sources and exogenous inputs, is propagated through appropriate lead-field or gain-matrices to generate observed sensor data. This spatial model has three key advantages. First, it is more appropriate than equivalent current dipole models, when real source activity is distributed locally within a cortical area. Second, the spatial degrees of freedom of the model can be specified and therefore optimised using model selection. Finally, the model is linear in the spatial parameters, which finesses model inversion. Here, we describe the distributed spatial model and present a comparative evaluation with conventional equivalent current dipole (ECD) models of auditory processing, as measured with EEG.

## Introduction

We have previously introduced a dynamic causal modelling (DCM) for event-related potentials and fields as measured with EEG and MEG (David and Friston 2003; David et al., 2005, 2006; Kiebel et al., 2006; Kiebel et al., 2007; Garrido et al., 2007). This extended the application of DCM beyond fMRI (Friston et al., 2003; Marreiros et al., 2008a) to cover EEG, MEG and local field potentials (Moran et al., 2007). However, all current DCMs model hemodynamic or electromagnetic signals as arising from a network of sources, where each source is considered to be a point process; i.e., an equivalent current dipole. In other words, the network is modelled as a *graph*, where sources correspond to *nodes* and conditional dependencies among the hidden states of each node are mediated by effective connectivity (known as *edges*). In this work, we replace the nodes with a distributed and continuous set of sources on the cortical surface. This provides a more realistic spatial model of underlying activity and, in the context of electromagnetic models, renders the DCM linear in its spatial parameters (c.f., Fuchs et al., 1999). The aim of this note is to describe this extension and compare it with models based upon point-sources or equivalent current dipoles (ECD). This model rests on the notions of mesostates (Daunizeau and Friston 2007) and anatomically informed basis functions (Phillips et al., 2002) but is motivated using neural-field theory (Amari, 1995; Jirsa and Haken, 1996; Liley et al., 2002).

DCM entails the specification of a generative model for an observed time-series and the inversion of this model to make inferences on model space and the parameters of each model. These inferences use the model evidence and posterior density of the parameters, respectively. In DCM, the underlying generative model is based on some state-equations (i.e., a state-space model) that describe the evolution of hidden states as a function of themselves and exogenous inputs (e.g., a stimulus function). The state-equations are supplemented with an observer function of the states to generate observed responses. By integrating the state-equation and applying the observer function, one obtains predicted responses. Under Gaussian assumptions about observation error these predictions furnish a likelihood model of observed responses. This likelihood model is combined with priors on the parameters to provide a full forward model of the data, which can be inverted using standard techniques (e.g., Friston et al., 2007a). These techniques generally rest on optimising a free-energy bound on the models log-evidence to approximate the posterior density of the model parameters.

In this work, we focus on the mapping from neuronal states to observed measurements at the sensors. We depart from equivalent current dipole models and employ an approximate neural-field model. Neural-field models describe electrocortical activity in terms of neuronal states (e.g. mean firing rate and post-synaptic membrane depolarisation) that are continuous over space (Amari 1995; Jirsa 1996; Liley 2002). This approach has shown how local and distal connectivity can interact to generate realistic spatiotemporal patterns of cortical activity that might underlie EEG rhythms (Nunez 1974) and their perceptual correlates, like visual hallucinations (Ermentrout and Cowan 1979). The patterns studied using neural-field models include *bumps* (transient clustering of activity) and *travelling waves* (which have been associated with synchronous discharges seen during epileptic seizures; Connors and Amitai 1993). These patterns are engendered by local (mesoscopic) connectivity. However, several authors have pointed out the importance of large-scale (macroscopic) connectivity in stabilizing local spatiotemporal dynamics (Jirsa and Kelso 2000; Breakspear and Stam 2005; Qubbaj and Jirsa 2007; Hutt and Atay 2005).

In this paper, we use theoretical results from neural-field theory, which combine mesoscopic and macroscopic connectivity, to model M/EEG. Specifically, we approximate the neural-field description of electrocortical activity with a set of distributed and continuous cortical sources that behave as standing-waves with compact local support. These standing-waves are coupled by temporal dynamics and follow from a truncated space-time decomposition of the solution of the underlying neural-field equations. The aims of this note are to (i) describe this neural-field DCM, (ii) compare it with established equivalent current dipole variants and (iii) to provide a framework for more realistic neural-field DCMs. We discuss these models in relation to the subtle balance between their face validity and identifiability.

In the first section of this paper, we derive a standing-wave approximation to the neural-field formulation and the ensuing parameterization of the DCM for distributed responses. In the second section, we present a comparative evaluation of DCMs based upon ECDs and distributed sources. We compare these models in terms of their relative log-evidence using a multi-subject EEG dataset, acquired during an auditory mismatch negativity paradigm. We conclude with a discussion of the benefits and potential uses of DCM for distributed responses.

## DCM for distributed responses

In this section, we approximate a neural-field description of electrocortical activity with local standing-waves. We then combine the ensuing spatial model with the temporal state-space models used in previous DCMs for event-related responses (David and Friston 2003; David et al., 2005, 2006; Kiebel et al., 2006, 2007; Garrido et al., 2007). This combination provides a full spatiotemporal DCM for distributed responses, which can be fitted or inverted in the usual way.

### From neural-fields to standing-waves

#### Neural-fields and mesoscopic modelling

We start with a description of the dynamics of a single neuron within an ensemble of neurons. These dynamics can be modelled as a temporal convolution of the average (mean-field) firing of the local population that is seen by the neuron:

(1) $$\begin{array}{l} x_{j}^{\left(i\right)}\left(t\right)=\int G\left(t-t\prime \right)\left({\langle H\left(x_{j}^{\left(i\right)}\left(t\right)-\theta \right)\rangle }_{j}+\gamma_{iu}u\left(t\right)\right)dt\prime \\ G\left(t\right)=\{\begin{array}{cc} \gamma \frac{t}{\kappa }\exp\left(-\frac{t}{\kappa }\right) & t\geq 0\\ 0 & t<0\text{.} \end{array} \end{array}$$

Here, *x*_*j*_^(*i*)^(*t*) is the post-synaptic membrane potential (PSP) of the *j*-th neuron in the *i*-th population; *G* is the alpha-kernel; *H* is the Heaviside function that models firing above depolarisation threshold *θ*; *κ* is a lumped rate-constant and *γ* controls the maximum post-synaptic potential. Eq. 1 assumes that any neuron senses all the neurons in the population it belongs to. This means endogenous input (from this population) can be written as the expected firing rate over that population (cf., Marreiros et al., 2008c). Here exogenous input (from another population or stimulus-bound subcortical input) is modelled as an injected current *u* scaled by the parameter *γ*_*iu*_. Eq. 1 can be reformulated in terms of an ODE (cf., David and Friston, 2003):

(2) $${\ddot{x}}_{j}^{\left(i\right)}-\kappa^{2}\gamma \left({\langle H\left(x_{j}^{\left(i\right)}-\theta \right)\rangle }_{j}+\gamma_{iu}u\right)+2\kappa {\dot{x}}_{j}^{\left(i\right)}+\kappa^{2}x_{j}^{\left(i\right)}=0\text{.}$$

**Fig. 1:**
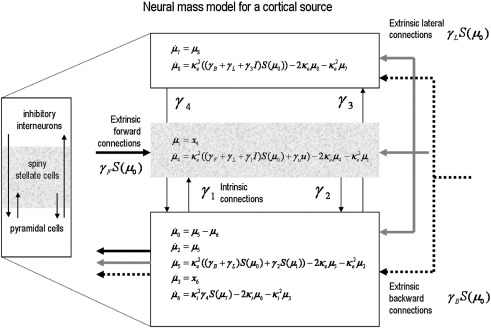
Neural-mass model. This figure depicts the schematic cytoarchitectonics of a cortical source, along with the differential equations used to model the dynamics of each of the three subpopulations (pyramidal, spiny stellate and inhibitory interneurons). These subpopulations have been assigned to granular and agranular cortical layers, which receive forward and backward connections, respectively. Here we have expressed the second-order ODEs in the text with pairs of first-order ODEs. This clarifies how the coupling parameters mediate influences among and between sources. Note that the infragranular population comprises two subpopulations (one excitatory and the one inhibitory). Source or region-specific superscripts have been dropped here for clarity.

Given Eq. 2 we can now model the dynamics of the population mean PSP by taking its expectation over neurons $\mu^{\left(i\right)}={\langle x_{i}^{\left(i\right)}\rangle }_{j}$ (Marreiros et al., 2008b):

(3) $$\begin{array}{c} {\ddot{\mu }}^{\left(i\right)}+2\kappa {\dot{\mu }}^{\left(i\right)}+\kappa^{2}\mu^{\left(i\right)}=\kappa^{2}\varsigma^{\left(i\right)}\\ \varsigma^{\left(i\right)}=\gamma_{iu}u+\sum_{j}\gamma_{ij}S\left(\mu^{\left(j\right)}\right)\\ S\left(\mu^{\left(j\right)}\right)=\frac{1}{1+\exp\left(-\rho \left(\mu^{\left(j\right)}-\theta \right)\right)} \end{array}$$

where *μ*^(*j*)^ corresponds to the mean PSP in each population *j* sending exogenous input. This is a conventional neural-mass model that effectively applies a linear synaptic (alpha) kernel to input—*ς*^(*i*)^, from other populations. This input is a nonlinear (sigmoid) function of depolarisation (Jansen and Rit 1995), which can be thought of as the cumulative probability distribution of PSPs over the population sending afferent signals. See Fig. 1, which shows the explicit form of the state-equations for a cortical source containing three fields or populations.

In DCM, a cortical source is typically modelled using three neuronal subpopulations corresponding roughly to spiny stellate *input* cells (in the granular layer) intrinsic inhibitory interneurons (assigned to the supragranular layer) and deep pyramidal *output* cells in the infragranular layer. The connectivity within (intrinsic) and between (extrinsic) sources conforms to the laminar rules articulated in Felleman and Van Essen (1991). This is implicitly modelled in Eq. 3 through the mixture of exogenous and endogenous inputs *ς* that depends on the connectivity or coupling parameters—*γ_ij_*. This sort of neural-mass model has been used to emulate electrophysiological recordings (e.g. Jansen and Rit 1995; Wendling et al., 2000; David et al., 2005) and as a generative model for event-related potentials in DCM (David et al., 2006).

However, these neural-mass models are not formulated to model spatially extended cortical regions (a square centimeter or so); they model the states of point processes, typically one macrocolumn (about 10,000 neurons, or a square millimeter of cortex; Breakspear and Stam, 2005). Neural-field models are important generalizations of neural-mass models, which account for the spatial spread of activity, through local connectivity between macrocolumns. In these models, states like the PSP of each cortical layer can be regarded as a continuum or *field*, which is a function of space *r* and time: *μ*^(*i*)^(*t*) → *μ*^(*i*)^(**r**,*t*). This allows one to formulate the dynamics of each field in terms of partial differential equations (PDE). These are essentially wave-equations that accommodate lateral interactions among neural-masses (e.g., cortical columns). Key forms for neural-field equations were proposed and analyzed by Nunez (1974) and Amari (1975). Jirsa and Haken (1996) generalized these models and also considered delays in the propagation of spikes over space. The introduction of delays leads to dynamics that are reminiscent of those observed empirically. Typically, neural-field models can be construed as a spatiotemporal convolution that can be written in terms of a Green function (see e.g. Jirsa et al., 2002):

(4) $$\begin{array}{c} \mu^{\left(i\right)}\left(r\text{,}t\right)=\int G\left(r-r\prime \text{,}t-t\prime \right)\varsigma^{\left(i\right)}\left(r\prime \text{,}t\prime \right)dt\prime dr\prime \\ G\left(r-r\prime \text{,}t-t\prime \right)=\delta \left(t-t\prime -\frac{1}{c}\left|r-r\prime \right|\right)\frac{1}{\gamma }\exp\left(-\frac{1}{\gamma }\left|r-r\prime \right|\right)\text{.} \end{array}$$

Here, *G* is a Green function (modelling mesoscopic lateral connectivity), |**r** − **r**′| is the distance between **r** and **r**′, *c* is the speed of spike propagation, *γ* controls the spatial decay of lateral interactions (within a neural-field) and, as above, the input *ς*^(*i*)^ models both the effective connectivity between the neural-fields of different populations or layers. Eq. 4 is formulated as a simple convolution^1^; the corresponding second-order equations of motion are the neural wave-equations (see Appendix 1):

(5) $$\left(\frac{\partial^{2}}{\partial t^{2}}+2\kappa \frac{\partial }{\partial t}+\kappa^{2}-\frac{3}{2}c^{2}\nabla^{2}\right)\mu^{\left(i\right)}\left(r\text{,}t\right)=c\kappa \varsigma^{\left(i\right)}\left(r\text{,}t\right)$$

where *κ* = *c* */* *γ* and ▿^2^ is the Laplacian operator that returns the spatial curvature. Note the similarity in form of Eqs. 3 and 5. These sorts of models have been extremely useful in modelling spatiotemporally extended dynamics, which unfold on the cortical manifold (see Deco et al. (2008) for a recent review, Coombes et al. (2007) for a more informed derivation of 2D neural fields and Robinson et al. (1997) for a seminal analysis of the properties of coupled neural-fields).

#### Approximating the dynamics of neural-fields

In what follows, we will try to approximate the dynamics described by the partial differential equations above, with a system whose dynamics can be described with the ordinary differential equations used in neural-mass formulations. Using separation of variables, it is fairly easy to show (see Appendix 2) that the solution of the neural-field equations can be expressed as a superposition of spatiotemporal modes that can be factorised into spatial and temporal components. For the *i*-th field or population:

(6) $$\mu^{\left(i\right)}\left(r\text{,}t\right)=\sum_{k}v_{k}^{\left(i\right)}\left(t\right)w_{k}^{\left(i\right)}\left(r\right)\text{.}$$

Here, *w*_*k*_^(*i*)^(*r*) is the *k*-th spatial mode or pattern and is the solution to the eigenvalue problem ▿^2^*w_k_^(i)^* + *λ*_*k*_^(*i*)^*w*_*k*_^(*i*)^ = 0. Note that this eigenvalue problem has to satisfy Dirichlet boundary conditions, i.e. the spatial modes are zero at the edges of the cortical region supporting the neural field. The temporal expressions of these modes are the eigenfunctions *v*_*k*_^(*i*)^(*t*) of the field, which obey the following second-order ODE:

(7) $${\ddot{v}}_{k}^{\left(i\right)}+2\kappa {\dot{v}}_{k}^{\left(i\right)}+\left(\kappa^{2}-\frac{3}{2}\lambda_{k}^{\left(i\right)}c^{2}\right)v_{k}^{\left(i\right)}=c\kappa \varsigma_{k}^{\left(i\right)}\left(t\right)$$

where the scalar input seen by each mode ς_*k*_^(*i*)^(*t*) is given by projecting the input field ς^(*i*)^ (**r**,*t*) onto that mode

(8) $$\varsigma_{k}^{\left(i\right)}\left(t\right)=\int w_{k}^{\left(i\right)}\left(r\right)\varsigma^{\left(i\right)}\left(r\text{,}t\right)dr\text{.}$$

This means the solution of the partial differential equations describing the spatiotemporal dynamics of neural-fields (Eq. 6) can be decomposed into spatial modes, *w*_*k*_^(*i*)^(**r**), weighted by the solutions of the coupled ODEs in Eq. 7, which describe the temporal dynamics of the neural-field.

We now want to simplify this description without compromising the dynamical repertoire of the model. Previous work on EEG/MEG source reconstruction suggests that most of the variance in EEG/MEG measurements can be accounted for by a set of temporally coherent and spatially extended cortical sources (see Daunizeau et al., 2006; Daunizeau and Friston, 2007; and Friston et al., 2007a,b). This coarse-grain description of electrocortical activity corresponds to a truncated spatiotemporal decomposition, in which each cortical region has just one spatial mode, whose activity is modulated over time (see also Jirsa et al. 2002; Wennekers 2008; and Robinson et al., 2001). Here, we motivate a related approximation based on equilibrium arguments. In the absence of exogenous input, each spatial mode decays at a rate that is proportional to $\kappa \left(1+\frac{c}{2}\sqrt{\frac{3}{2}\lambda_{k}^{\left(i\right)}}\right)$. This is important, because high propagation velocities *c*, will dissipate the spatial modes quickly, with the exception of the fundamental mode *w*_0_, which has a zero eigenvalue; *λ*_0_^(*i*)^ = 0. This means that, after a short period of time, the depolarisation of the *i*-th population or field will become a standing-wave; corresponding to fluctuations of the fundamental mode:

(9) $$w_{0}^{\left(i\right)}\left(r\right)\left({\ddot{v}}_{0}^{\left(i\right)}\left(t\right)+2\kappa {\dot{v}}_{0}^{\left(i\right)}\left(t\right)+\kappa^{2}v_{0}^{\left(i\right)}\left(t\right)\right)=c\kappa w_{0}^{\left(i\right)}\left(r\right)\varsigma_{0}^{\left(i\right)}\left(t\right)\text{.}$$

Here, *v*_0_^(*i*)^(*t*) describes the temporal evolution of this mode. Critically, these dynamics have exactly the same form as the neural-mass model; i.e., when *λ*_*k*_^(*i*)^ = 0, Eq. 9 is formally identical to Eq. 3. This suggests that we can model distributed responses using a single mode or pattern, whose fluctuations are coupled by the dynamics of conventional neural-mass models (see Appendix for details).

In summary, by ignoring all but the fundamental mode, we can model the spatiotemporal dynamics of each population or layer as fluctuations in a single spatial mode—*w*_0_^(*i*)^(**r**). Under this approximation, the dynamics of coupled populations become a simple system of coupled standing-waves, each of which behaves like a neural-mass. The temporal dynamics *v*_0_^(*i*)^(*t*) of these modes are exactly the same as neural-mass models, where the mean PSP is replaced by the eigenfunction:

(10) $$\begin{array}{c} {\ddot{v}}_{0}^{\left(i\right)}+2\kappa {\dot{v}}_{0}^{\left(i\right)}+\kappa^{2}v_{0}^{\left(i\right)}=c\kappa \varsigma_{0}^{\left(i\right)}\left(t\right)\\ \varsigma_{0}^{\left(i\right)}\left(t\right)=\gamma_{iu}u+\sum_{j}\gamma_{ij}S\left(v_{0}^{\left(j\right)}\right)\\ :=\int w_{0}^{\left(i\right)}\left(r\right)\varsigma^{\left(i\right)}\left(r\text{,}t\right)dr\text{.} \end{array}$$

These approximations allow us to relate neural-mass DCMs to more realistic neural-field models. From the perspective of neural-fields, neural-mass models correspond to an approximation, which is valid when the system is close to equilibrium; i.e. when the interactions between the modes do not drive the system into autonomous behaviour (bumps or travelling waves) and most modes decay quickly. This is typically assumed to be the case for event-related responses (ERPs), which are generally slow damped oscillatory responses to stimulation (e.g. Kiebel et al., 2007; Garrido et al., 2008). It would be possible to increase the number of modes per population or field to provide a more complete neural-field model; however, this is beyond the scope of the present work. Our model now comprises a set of neural-masses, whose dynamics modulate the expression of some unknown but fixed spatial modes. Next, we consider how these modes are modelled.

### The spatial model

Due to the Dirichlet constraints at the boundary of the cortical regions and local variations in cortical curvature, the fundamental mode **w**_0_^(*i*)^ of the Laplacian operator can have an arbitrary spatial profile. Therefore, we model it as a mixture of spatial basis functions, derived from the gain-matrix associated the cortical region:

(11) $$w_{0}^{\left(i\right)}=\sum_{n}U_{n}^{\left(i\right)}\beta_{n}^{\left(i\right)}\text{.}$$

Here, ***U***_*n*_^(*i*)^ are the spatial eigenvectors of the gain-matrix **L**^(*i*)^ associated with the set of vertices of the cortical mesh belonging to the *i*-th source or region, and *β*_*n*_^(*i*)^ are the unknown spatial parameters of our DCM. In addition, we assume that each cortical layer (neuronal population) within each region can contribute to the EEG/MEG signal measured at the sensors. This leads to the following DCM for distributed responses:

(12) $$y\left(t\right)=\sum_{i}L^{\left(i\right)}w_{0}^{\left(i\right)}\sum_{j}J_{j}v_{0}^{\left(ij\right)}\left(t\right)+ɛ\text{.}$$

where ***y***(*t*) is the column vector of instantaneous EEG/MEG scalp measurements and **L**^(*i*)^ are the gain-matrices for the *i*-th region. The unknown relative contributions *J*_*j*_ of the eigenfunctions *v*_0_^(*ij*)^(*t*) of the *j*-th population in the *i*-th cortical region are assumed to be the same for all regions. Note that the fundamental mode is the same for all populations within the same region because it depends only on the geometry of the regional cortical manifold. The free-parameters of the DCM now comprise the spatial parameters ϑ⊃{β,J} (Eq. 11) and the neuronal parameters ϑ⊃{κ,γ} of the ODE (Eq. 10); these encode synaptic rate-constants and coupling parameters, respectively.

The decomposition of the spatial mode into the principal components of the gain-matrix (Eq. 11) suppresses redundancy in the spatial model; in the sense that spatial modes that cannot be seen by the sensors are precluded. In our implementation, the user specifies the coordinates of the sources comprising the network in canonical space (Mattout et al., 2007; Talairach and Tournoux 1988). The mesh points constituting each source are then identified automatically as those points lying within a sphere centred on the prior source location. We then take the first eight eigenvectors of **L**^(*i*)^**L**^(*i*)*T*^ to produce the spatial basis functions **U**^(*i*)^. The lead-fields are computed using BrainStorm (http://neuroimage.usc.edu/brainstorm/), after co-registering the channel locations to a subject-specific canonical mesh (Mattout et al., 2007). This involves warping a template mesh (in canonical space) to match the anatomy of each subject; so that individual differences in anatomy are accommodated but the mapping between subject-specific meshes and canonical space is preserved. The warping uses standard nonlinear spatial normalisation tools in SPM (http://www.fil.ion.ucl.ac.uk/spm).

### Model inversion

Model inversion proceeds using standard variational techniques under the Laplace assumption as described in previous communications (e.g., Friston et al., 2007a). The products of this inversion are a free-energy approximation to the model's log-evidence ln *p*(*y*|*m*) and an approximating posterior density on the model parameters, *q*(*ϑ*) = *N*(*μ*_*ϑ*_,Σ_*ϑ*_), where *μ*_*ϑ*_ is the posterior expectation and Σ_*ϑ*_ is the posterior covariance. This inversion entails the computation of the gradients and curvatures of the log-likelihood function, provided by the likelihood model (Eq. 11). This involves computing the derivatives of the predicted response with respect to model parameters; i.e., integrating the neuronal state-equations to see how they respond to stimulus-related input (a parameterized Gaussian bump-function of peristimulus time) and then repeating this under small perturbations of the parameters. Critically, the computation of the derivatives with respect to the spatial parameters can be simplified greatly if the response is linear in the parameters. This is the case for the distributed source model, under which

(13) $$\begin{array}{c} \frac{\partial y}{\partial \beta_{n}^{\left(i\right)}}=L^{\left(i\right)}U_{n}^{\left(i\right)}\sum_{j}J_{j}v_{0}^{\left(ij\right)}\\ \frac{\partial y}{\partial J_{j}}=\sum_{i}L^{\left(i\right)}\sum_{n}U_{n}^{\left(i\right)}\beta_{n}^{\left(i\right)}v_{0}^{\left(ij\right)}\text{.} \end{array}$$

This is not the case for the DCMs based on ECDs, which have nonlinear observer functions with six spatial parameters (encoding the location of the source and its orientation). With the present spatial model, we only have to integrate the system once, given the current estimate of the neuronal parameters, *γ*, *κ* to get *v*_0_^(*ij*)^. These are then used to compute the gradients in Eq. 13. This speeds up the iterative variational scheme, as compared to the conventional DCMs based on ECDs.

In what follows, we will focus on model comparison under ECD and distributed spatial models, using the same temporal model. We will use Monte Carlo simulations to assess sensitivity and real ERP data to compare the spatial models in terms of their evidence. A difference in log-evidence of three is usually considered significant; because this suggests a relative likelihood of 20:1. Under flat priors on the models, this means that one can be 95% confident that one model is better than the other.

## Comparative evaluations

### A sensitivity analysis

We are primarily interested in making inferences about the connectivity of the network generating data (encoded by *γ*_*ij*_). However, the estimation of these parameters will be sensitive to the specification of the generative model (e.g. the prior position of the sources). In this section, we quantify the relative robustness (if any) of the DCM for distributed responses, relative to ECD models, to variations of the generative model. To assess robustness we compared the changes in the posterior estimates of the neuronal parameters (i.e. synaptic efficacies and rate-constants, which are common to both models) when changing the prior or likelihood of the DCM.

**Fig. 2:**
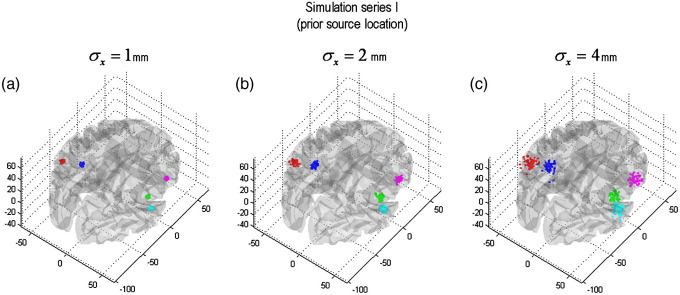
Simulation series I: changing the prior locations. This figure depicts the three levels of perturbation to the prior location of the sources, as quantified by the standard deviation *σ*_*x*_ of the distance between the true (simulated) position of the sources and the location that has been used to specify the DCM. (a) The 50 random samples of prior location of the five sources (for *σ*_*x*_ = 1mm). (b) Id for *σ*_*x*_ = 2mm. (c) Id for *σ*_*x*_ = 4mm.

**Fig. 3:**
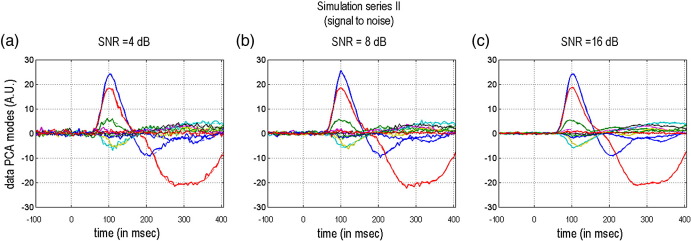
Simulation series II: changing signal to noise. This figure shows the three levels of measurement noise in the synthetic data as quantified by the signal-to-noise ratio (SNR). (a) One sample of a synthetic data set (projected onto the sixteen spatial components of a PCA decomposition), at SNR = 4 dB. (b) at SNR = 8 dB. (c) at SNR = 16 dB.

To equate the degrees of freedom (number of parameters) in both models, we used six spatial basis functions to model each mode (ECD models have six spatial parameters encoding the location and orientation of each dipole). First, we computed the predictions $\hat{y}$ after fitting two DCMs (ECD and distributed) to real mismatch negativity event-related potentials (ERPs) (see next section). This produced two sets of data, generated by ECD and distributed DCMs, with different but known neuronal parameters. These were then used as synthetic data for a series of DCM inversions, as follows:

We ran two sets of simulations. In series I, we perturbed the prior mean of the [five] source locations. We examined three levels of perturbation: *σ*_*x*_ ∈ {1,2,4} mm, where *σ*_*x*_ was the standard deviation of random Gaussian perturbations to the prior mean (see Fig. 2). In series II, we perturbed the likelihood by adding Gaussian noise to the data; using three signal-to-noise ratios: SNR ∈ {4,8,16} dB. SNR is defined as: SNR = 10 ln var ($\hat{y}$)/var(*ɛ*) (see Fig. 3). We used 50 Monte Carlo samples for both series.

Given the true parameters of the generative model and their posterior estimator, we can evaluate the squared error loss:

(14) $$\mathrm{SEL}\left(\vartheta \right)=\sum_{i}{\left(\vartheta_{i}-{\hat{\vartheta }}_{i}\right)}^{2}$$

where ${\hat{\vartheta }}_{i}$ is the *i*-th neuronal parameter. The SEL is a standard estimation error measure, whose posterior expectation is minimized by the mean of the posterior density. This means that using the posterior mean as an estimator $\hat{\vartheta }={\langle \vartheta \rangle }_{q}$ of unknown *ϑ* is optimal with respect to squared error loss.

**Fig. 4:**
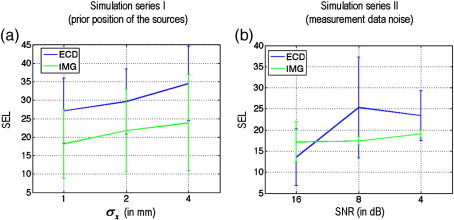
Monte Carlo simulation results-squared error loss. Both graphs show the squared error loss (SEL) as a function of the level of prior dislocation and noise (error bars correspond to one standard deviation). (a) SEL as a function of perturbation on the prior location of the sources—*σ*_*x*_. (b) SEL as a function of signal to noise-SNR. Except for the highest level of SNR (16 dB), where both ECD and distributed DCMs behave similarly, the spatially distributed DCM is consistently better than its ECD variant.

**Fig. 5:**
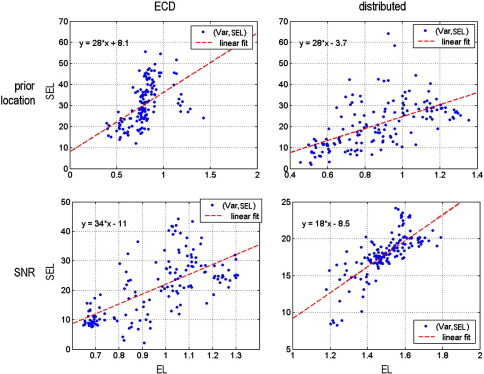
Monte Carlo results—posterior confidence. Both graphs show the expected (*x*-axis) versus the observed (*y*-axis) squared error loss (SEL) for both series of simulations and spatial variants of the DCM. Top: changes in prior locations. Bottom: changes in signal to noise. Left: ECD-DCM. Right: distributed DCM. Although there is an order of magnitude difference between the predicted and observed SEL, they are strongly correlated. Note the increase in correlation for distributed DCMs over ECD-DCMs (bottom right).

We investigated how the SEL changed as a function of prior location and SNR, for both the ECD and the distributed solutions (see Fig. 4). It can be seen that the distributed DCM is consistently better than its ECD homologue, except at the highest SNR (16 dB), where both models show the same squared error loss. In short, the estimation error on the neuronal parameters, as measured by the squared error loss is much smaller for the distributed DCM, which is less sensitive to noise and inaccurate priors than its ECD variant. In addition, we evaluated the quality of posterior confidence intervals: under the Laplace approximation; *q*(*ϑ*) = *N*(*μ*_*ϑ*_,Σ_*ϑ*_), this reduces to assessing the accuracy of the posterior covariance in relation to the SEL, since;

(15) $$\mathrm{EL}\left(q\right)={\langle \mathrm{SEL}\left(\vartheta \right)\rangle }_{q}=tr\left(\Sigma_{\vartheta }\right)\text{.}$$

where the expected loss EL(*q*) is the Bayesian estimator of SEL (see Robert, 1992). This equivalence means we can assess the posterior covariance in terms of the relationship between the expected and the sampled SEL; for both the ECD and distributed solutions. A good correlation between the expected EL(*q*) = *tr*(Σ_*ϑ*_) and observed SEL means that the inference scheme is self-consistent; i.e., it adapts its level of confidence in proportion to the real (observed) estimation error. Fig. 5, shows the expected versus the observed SEL for both series of simulations and DCMs. Although there is an order of magnitude difference between the predicted and the observed SEL, they are strongly correlated. In addition, the correlation between EL(*q*) and SEL under different levels of noise is significantly higher for the distributed DCM.

In summary, the DCM of distributed responses is more robust to violations of priors and levels of noise; furthermore, it is more self-consistent in that the observed and expected estimation loss is more tightly coupled, relative to ECD models. We now turn to empirical comparisons, using the relative evidence for both models in real data.

### Model comparisons using EEG data

In this section, we apply both ECD and distributed DCMs to the grand-mean responses from an eleven-subject auditory mismatch negativity study (Garrido et al., 2007). The term ‘mismatch negativity’ (MMN) describes an evoked response component elicited by the presentation of a rare auditory stimulus in a sequence of repetitive standard stimuli (Näätänen, 2003). The rare stimulus typically causes a more negative response. The difference between deviant and standard tone reaches a minimum at about 100 ms, and exhibits a second minimum later between 100 and 200 ms.

**Fig. 6:**
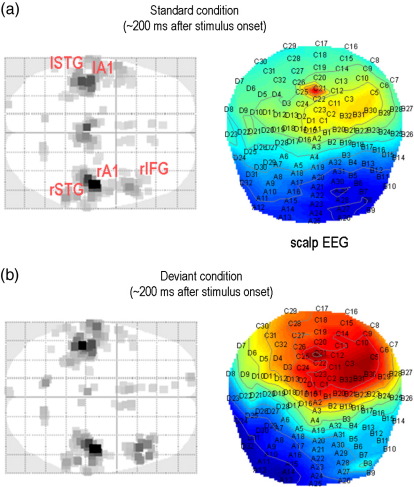
Mismatch negativity study: scalp data and source reconstructions. The mismatch negativity (MMN) is the pattern elicited when contrasting a standard condition (repeated high-pitched tones) with a deviant condition (sparse low-pitched tones). This figure shows both the scalp topography and the corresponding source reconstructions at the time of the maximum difference (approx. 200 ms after onset). (a) Standard condition: the maximum intensity projection (MIP) on the source reconstruction shows five key sources: right/left primary auditory cortex (A1), right/left superior temporal gyrus (STG) and right inferior frontal gyrus (IFG). (b) Deviant condition: the MIP shows the same five sources (with different amplitudes).

**Fig. 7:**
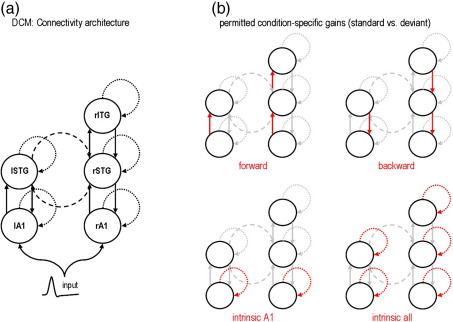
Mismatch negativity study: DCM architectures. The five sources identified by source reconstruction MIP (see Fig. 6) were used to construct a DCM network as follows: (a) both primary auditory sources were coupled with forward and backward connections to ipsilateral STG sources. The latter were reciprocally connected through lateral connections, and right STG was coupled with forward and backward connections to rIFG. Within this graph, we compared eleven models, corresponding to different combinations of connectivity changes between the standard and the deviant conditions of the MMN paradigm. These condition-specific changes are depicted in (b): four sets of connections were allowed to change: forward connections, backward connections, intrinsic connections for bilateral A1, and all intrinsic connections. We then derived eleven DCMs from combinations of these four sets; “F”, “B”, “FB”, “FI”, “BI”, “FBI”, “FA”, “BA”, “FBA”, and “0” (see Table 1).

We first performed a conventional imaging source reconstruction to specify the underlying neuronal network, in terms of the number and prior expectations of source locations (Friston et al., 2007b). Fig. 6 shows the results of a source reconstruction for the first subject and highlights the prior source locations selected for the DCM analyses. This network is shown in Fig. 7a, which includes the extrinsic (between source) connections (cf. Garrido et al., 2007). In brief, we allowed for forward and backward connections between an early bilateral auditory (rA1 and lA1) source and bilateral superior temporal gyrus (rSTG and lSTG) areas, as well as forward and backward connections between the right STG and a source located in the inferior frontal gyrus (rIFG). We also included transcallosal lateral connections between the STG sources.

The conventional understanding of the MMN rests on change-sensitive neuronal populations. In Kiebel et al., (2007), we considered two hypotheses, which explain the MMN either by adaptation or within a predictive coding framework (Friston, 2005; Garrido et al., 2008). We have shown that hypotheses like these can be formulated and tested using DCM, by allowing connections to change between the deviant and the standard conditions. In particular, we can test hypotheses about the mechanisms underlying the MMN by modelling the response evoked by a deviant using the same parameters as for the standard response, except for a gain in selected connections. Here, we repeat this analysis using both spatial variants of DCM.

Table 1 shows the different architectures we considered in terms of which connections were allowed to change (from the deviant to standard conditions). In brief, these different models correspond to different explanations for the MMN: the adaptation hypothesis (change in intrinsic connections) and the predictive coding hypothesis (change in intrinsic and extrinsic connections). We refer the interested reader to Kiebel et al., (2007):

Four sets of connections were allowed to change between the deviant and the standard conditions (see Fig. 7b); ‘forward’ implies permissible changes in all forward connections; ‘backward’, all backward connections; intrinsic A1′, changes in connectivity intrinsic to A1 and ‘intrinsic all’, all intrinsic connections. We then constructed eleven DCMs from combinations of these four basic differences, namely; “F”, “B”, “FB”, “FI”, “BI”, “FBI”, “FA”, “BA”, “FBA”, and “0” (see Table 1). The last model precluded any changes between the two conditions and constitutes a null model.

**Fig. 8:**
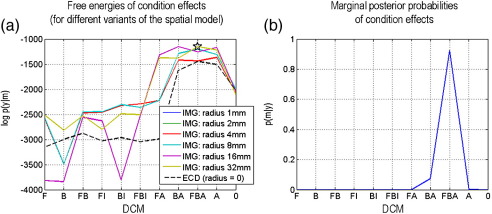
Mismatch negativity study: Bayesian model comparison results. Bayesian model comparison was applied to an 11 × 7 factorial model space. Eleven condition effects (see Fig. 6 b) and 7 spatial variants of each DCM (6 distributed DCMs, with different cortical regions (sphere radii ∈ 1, 2, 4, 8, 16, and 32 mm) and one ECD-DCM. (a) Free-energies (log-evidences) for each of the 11 × 7 models. The star indicates that the best of all DCMs is a distributed FBA model (in which all connections were allowed to change) with the largest region. (b) Marginal posterior probabilities of the eleven DCMs (marginalising over spatial models).

In these comparative analyses, we also investigated the effect of changing the spatial support of the cortical regions in the distributed DCMs. This was achieved by varying the radius (1, 2, 4, 8, 16 and 32 mm) of the sphere (centred on the prior location), which defines the mesh vertices in each cortical region. We used the free-energy approximation to the log-evidence to compare the 11 × 7 = 77 models; eleven DCMs with seven spatial models (six distributed models with different spheres and one ECD model). The ensuing log-evidences are shown in Fig. 8. For almost all DCMs, the ECD models were significantly less likely than distributed DCMs (max *F*_distributed_ − max*F*_*ECD*_ = 294.8). Note that this model comparison automatically accommodates differences in model complexity. These differences were small because the ECD and the distributed and ECD-DCMs had the same number of parameters.

When the sphere radius in the distributed DCM is reduced, the DCMs have very similar log-evidences (Fig. 8a). Note also that the model evidence of the best DCM (FBA) for distributed DCM with small (1, 2, and 4 mm) spheres and the ECD model are very close to each other. This means that these spatial models converge when inverting a well-specified neuronal model. In other words, there is no significant difference in model evidence between ECD and small patches, since the latter are approximated well by a single dipole. Fig. 8 also shows the marginal posterior probabilities of the eleven DCMs; marginalising over all spatial variants. This integrates out dependency on the spatial parameters and replicates the finding of Garrido et al., (2007) that the most plausible DCM seems to combine changes in forward, backward and intrinsic connections. For this DCM, there was strong evidence that the distributed DCM (all radii) was a better model than the ECD equivalent.

## Discussion

We have described a variant of dynamic causal modelling for event-related potentials or fields as measured with EEG and MEG. We motivated this DCM as an approximation to a continuous neural-field model, using a mixture of overlapping patches, with compact spatial support, on the cortical surface. Time-varying activity in this mixture, caused by activity in other sources and experimental inputs, is propagated through appropriate lead-field or gain-matrices to generate observed channel data. In comparison to ECD variants of DCM, this distributed DCM has three advantages; it has greater face validity, the degrees of freedom of the spatial model can be specified (and therefore optimised using model selection) and the model is linear in the spatial parameters (which finesses computational load). Both our simulations and the application to an EEG auditory mismatch negativity dataset demonstrated the superiority of distributed DCMs, when compared to their ECD homologues.

The greater face validity of spatially distributed DCMs is similar to that of imaging source reconstruction solutions, when compared to ECD-like solutions: the spatial extent of each regional source must be modelled properly when inverting such models (see below). Furthermore, the neural-mass models we use (Jansen and Rit 1995) were designed originally to model mesoscopic electrocortical activity, at a spatial scale finer than that of EEG/MEG. Using simple approximations of neural-field models, we have proposed a simple modification of neural-mass models that render them able to emulate macroscopic spatiotemporal dynamics. Specifically, these modifications allow us to account for the spatial deployment of sources, which appears to be necessary to explain EEG/MEG data (see MMN results section).

Although not pursued here, the number of basis functions or different sizes of cortical regions could be optimised. One would repeat the inversion using different basis functions and evaluate the model evidences (as for the analysis of cortical sources in Fig. 8). This would allow one to optimise the degrees of freedom of the spatial model, in relation to the spatial information supported by the data; similarly for the size of the cortical patches used to model source-specific activity.

Note that there is a formal link between the spatially distributed DCM proposed in this work and EEG/MEG source reconstruction techniques (see e.g. Daunizeau et al., 2006; Friston et al., 2007b). The key difference between these two approaches rests on the formal constraints used by DCM. These constrain the temporal expression of source activity to conform to a biologically plausible time-course (Scherg and Von Cramon 1985). The interpretation of a DCM analysis is not usually concerned with the spatial profile of source activity but focuses on the coupling parameters and how they change with experimental manipulations. However, it is interesting to regard the DCM inversion as a biophysically and neurobiologically informed imaging source reconstruction (see Kiebel et al., 2006). In other words, one can regard the Bayesian inversion of spatially distributed DCM as a generalisation of classical forward model inversion used to reconstruct source activity from observed EEG or MEG data. The only difference between classical inversion and DCM is that the source activity has to conform to a biophysically plausible model. Generally, this model entails interactions among sources so that activity in one source is caused by activity in others. Classical forward models focus exclusively on the spatial observer function of the hidden states and ignore formal constraints on the temporal expression of source activity. The resulting spatial models are either ECD-based models or distributed source models of the sort used in image reconstruction (Baillet and Garnero 1997; Pascual-Marqui 2002; Phillips et al., 2005). Exactly the same distinction between ECD and distributed reconstructions can be applied in the context of DCM. In this note, we have described a distributed spatial model that complements existing ECD dynamic causal models.

In the future, it is possible that DCMs will be based on models that are closer to full neural-field models. These models might be more appropriate for EEG and MEG data because they account for continuous lateral interactions within each cortical region. Neural-field models can generate time-dependent dynamics that are expressed as bumps or propagating waves over the cortical surface. In this work, we truncated our space-time decomposition to the fundamental mode (a zero-order approximation). As a consequence, the neural-fields behave as interacting standing-waves; i.e. regionally specific invariant patterns of activity oscillating in response to mutual influence. This space-time separation is a simplified variant of the sort of the spatiotemporal behaviours that could be obtained using a more realistic wave-equation (c.f. Eq. 6). Our zero-order approximation could be relaxed to increase the complexity of the neural-field model. This can be done by including more modes (see Eqs. 7 and 8 and Appendix 2). This would allow one to replace a full PDE to a set of coupled ODEs.

Two additional comments should be made: first, the derivation of the 2D neural field PDE relies on the assumption that lateral (isotropic) interactions are deployed over a small spatial scale (see Appendix 1). As a consequence, only long spatial wavelengths (relative to the spatial decay of lateral interactions) can be expressed in the 2D cortical neural field. This means that mesoscale phenomena like patchy feature maps (e.g. orientation preference or ocular dominance) in V1 might not be captured accurately (see Bressloff 2003 for a recent discussion of isotropic connectivity and Coombes et al., 2007 for an extension of the long-wavelength approximation to *patchy propagators*).

Second, we motivated our standing wave (fundamental mode) approximation to the neural field by noting that at high propagation velocity, higher harmonics will dissipate quickly. This is consistent with more realistic models (including axonal propagation), which also suggest that higher harmonics are damped more heavily (Nunez 1995). However, our standing wave approximation to experimentally manipulated (excited) neural fields is different in nature from the emergence of *global* standing-waves as proposed in Nunez and Srinivasan (2006). The latter global waves are thought to underlie global coherence of cortical activity in the absence of stimulation (e.g. eyes-closed resting alpha-band activity). Global standing-waves can be thought of as a resonance phenomenon, whose wavelength is related to the size of the brain. Nunez points out that mental tasks “enhance cell assembly activity [i.e. functional segregation], thereby reducing global field behaviour”. This is in contradistinction to the present work, which postulates that local standing-waves emerge from the interaction of segregated neural ensembles. According to this view, segregation is *necessary* for the standing-waves to emerge, in the sense that it prevents activity spreading over the cortical mantle. In turn, this makes extrinsic functional integration (i.e. between region top-down and bottom-up effects, as opposed to within region lateral interactions) the principal mechanism responsible for sustained large-scale cortical activity.

## Software note

All the routines and ideas described in this paper can be implemented with the academic freeware SPM8 (http://www.fil.ion.ucl.ac.uk/spm).

## Figures and Tables

**Table 1:** Condition-specific effects (standard versus deviant): gain in coupling strength.

	Forward	Backward	Intrinsic A1	Intrinsic all
F	X			
B		X		
FB	X	X		
FI	X		X	
BI		X	X	
FBI	X	X	X	
FA	X			X
BA		X		X
FBA	X	X		X
A				X
0				

## Appendices

### Approximate 2D neural fields on the cortical manifold

Deriving the partial differential equation describing the spatiotemporal dynamics of neural fields from the underlying integrodifferential equation is a difficult problem because: (i) even on a Euclidean space, the Fourier analysis of the 2D neural field is not exact and (ii) on curved (Riemannian) manifolds, Euclidean distance measures do not apply. In this appendix, we discuss approximate solutions to these problems.

First, we consider the neural field unfolding on a planar surface tangential to the cortical manifold. Let **r** = (*r*,*θ*) denote the 2D position (in polar coordinates) on this Euclidean space. Recall that the spatiotemporal convolution that operates on the input *ς*(*r*,*t*) is given by Eq. 4

(A1) $$\begin{array}{c} \mu \left(r\text{,}t\right)=G\left(r\text{,}t\right)*\varsigma \left(r\text{,}t\right)\\ G\left(r\text{,}t\right)=\delta \left(t-\frac{1}{c}r\right)\underset{\text{lateral interaction}}{\underset{︸}{\frac{1}{\gamma }\exp\left(-\frac{1}{\gamma }r\right)}} \end{array}$$

where ⁎ denotes convolution and *G* is the convolution kernel, whose spatial scale is controlled by the decay of lateral interactions *γ*. Therefore, if the Fourier transform $\tilde{G}\left(k\text{,}\omega \right)$ of *G* can be represented in the rational form $\tilde{G}\left(k\text{,}\omega \right)$ = *R*(*k*^2^,*iω*) / *P*(*k*^*2*^,*iω*), we have *P*(*k*^2^,*iω*)*G*(*k*,*ω*) = *R*(*k*^2^,*i*ω)*ζ*(*k*,*ω*). By identifying *k*^2^ ↔ − ▿^2^ and iω↔∂/∂t, an inverse Fourier transform will yield the PDE in terms of spatial and temporal derivatives (see Coombes et al., 2007). Given the functional form of the lateral interactions, Liley et al. (2002) proposed an expansion of *P*(*k*^2^,*iω*) near *k* = 0, yielding the “long-wavelength” approximation:

(A2) $$\left(\frac{\partial^{2}}{\partial t^{2}}+2\kappa \frac{\partial }{\partial t}+\kappa^{2}-\frac{3}{2}c^{2}\nabla^{2}\right)\mu \left(r\text{,}t\right)=c\kappa \varsigma \left(r\text{,}t\right)$$

where *κ* = *c* / *γ*. This expression is very close but not identical to that obtained more simply (and exactly) for 1D neural fields (e.g. Deco et al., 2008). The long-wavelength approximation basically implies that *k* << 1/*γ*, i.e. *Δr* ≫ *γ*, where *Δr* is the typical spatial wavelength. This is not a critical assumption when modelling EEG scalp data; since the head volume conductor acts as a low-pass spatial filter such that scalp potentials are dominated by the long-wavelength components engendered by cortical sources (Nunez and Srinivasan 2006).

The PDE (A2) derives from the spatially invariant form of the Green function above (A1), which, on a 2D Riemannian manifold is:

(A3) $$G\left(r\text{,}r\prime \text{,}t\right)=\delta \left(t-\frac{d\left(r\text{,}r\prime \right)}{c}\right)\frac{1}{\gamma }\exp\left(-\frac{d\left(r\text{,}r\prime \right)}{\gamma }\right)\text{,}$$

where **r**(resp. **r**′) is the position on the cortical surface of the target (resp. source) neuron of the neural field, and *d*(**r**,**r**′) is the distance metric on the cortical manifold. Note that the cortical manifold more precisely, each hemisphere) is homotopic to a sphere, which means that its metric is well-behaved and that local geographic (angular) coordinates can be defined on the cortical mantle (Toro and Burnod 2003). This implies that a patch of the cortical mantle is homotopic to an open set in *R*^2^, where there is a bijective mapping from the angular coordinates to the Euclidean polar coordinates above. If the fall-off distance *γ* is small compared to the inverse curvature (smoothness) of the manifold *Δ*, the Green function can be approximated by a spatially invariant convolution kernel:

(A4) $$G\left(r\text{,}r\prime \text{,}t\right)\overset{\gamma =\Delta }{\to }\delta \left(t-\frac{\left|r-r\prime \right|}{c}\right)\frac{1}{\gamma }\exp\left(-\frac{\left|r-r\prime \right|}{\gamma }\right)\equiv G\left(r-r\prime \text{,}t\right)\text{.}$$

This is because contributions from the manifold that diverge from the tangent surface (e.g. neighbouring sulci) will be negligible. Note that this “short-scale lateral connectivity” is required because the assumption of isotropic lateral interaction posits that the boundary effects are negligible far from boundaries. Moreover, it justifies the above “long-wavelength” approximation, in the sense that such a system can almost only resonate at long-wavelength harmonics (relative to *γ*). One can think of the short-scale lateral connectivity in a 1D neural field as a string of interacting (infinitesimal) elements (e.g., under tension). The energy required to excite the string is proportional to the frequency of its harmonics, which means that higher harmonics (short wavelengths) will decay quickly. These considerations mean that under “short-scale lateral connectivity”, the PDE obtained from the lateral interaction function above (A4) is approximately valid.

### Approximate solutions for neural-fields

The neuronal activity of a cortical layer or population can be modelled as a neural-field *μ*^(*i*)^(*r*,*t*) that satisfies the following partial differential equation (PDE):

(A5) $$\begin{array}{l} \left(\frac{\partial^{2}}{\partial t^{2}}+2\kappa \frac{\partial }{\partial t}-\frac{3c^{2}}{2}\nabla^{2}\right)\mu^{\left(i\right)}\left(r\text{,}t\right)=f^{\left(i\right)}\left(r\text{,}t\right)\\ f^{\left(i\right)}\left(r\text{,}t\right)=c\kappa \varsigma^{\left(i\right)}\left(r\text{,}t\right)-\kappa^{2}\mu^{\left(i\right)}\left(r\text{,}t\right) \end{array}$$

Here, *μ*^*(i)*^(**r**,*t*) is the field associated with the *i*-th population. In (A5), we have lumped input and decay terms into *f*^*(i)*^(**r**,*t*). Using separation of variables, we can express the solution of this PDE as:

(A6) $$\mu^{\left(i\right)}\left(r\text{,}t\right)=\sum_{k}v_{k}^{\left(i\right)}\left(t\right)w_{k}^{\left(i\right)}\left(r\right)$$

where the spatial modes *w*_*k*_^(*i*)^(**r**) are the solutions to the eigenvalue problem:

(A7) $$\nabla^{2}w_{k}^{\left(i\right)}+\lambda_{k}^{\left(i\right)}w_{k}^{\left(i\right)}=0\text{.}$$

Note that the self-adjoint property of the Laplacian operator ▿^2^ ensures the eigenvalues *λ*_*k*_^(*i*)^ are real and the spatial eigenvectors or modes *w*_*k*_^(*i*)^(**r**) are real and orthonormal

(A8) $$\int w_{k}^{\left(i\right)}\left(r\right)w_{k\prime }^{\left(i\right)}\left(r\right)dr=\delta_{kk\prime }\text{.}$$

This orthonormal property means we can express the eigenfunctions *v*_*k*_^(*i*)^(*t*) as

(A9) $$v_{k}^{\left(i\right)}\left(t\right)=\int w_{k}^{\left(i\right)}\left(r\right)\mu^{\left(i\right)}\left(r\text{,}t\right)dr\text{.}$$

If we differentiate (A9) with respect to time and use (A5) to eliminate ${\dot{\mu }}^{\left(i\right)}$, we obtain

(A10) $$\begin{array}{c} {\dot{v}}_{k}^{\left(i\right)}\left(t\right)={\int w}_{k}^{\left(i\right)}\left(r\right){\dot{\mu }}^{\left(i\right)}\left(r\text{,}t\right)dr\\ =\frac{1}{2\kappa }\left(-\int w_{k}^{\left(i\right)}\ddot{\mu }dr+\frac{3}{2}c^{2}{\int w_{k}^{\left(i\right)}\nabla }^{2}\mu^{\left(i\right)}dr+\int w_{k}^{\left(i\right)}fdr\right)\text{.} \end{array}$$

From the form of the solution (A6) and (A7), we have

(A11) $$\begin{array}{c} {\ddot{\mu }}^{\left(i\right)}\left(r\text{,}t\right)=\sum_{k}{\ddot{v}}_{k}^{\left(i\right)}\left(t\right)w_{k}^{\left(i\right)}\left(r\right)\\ \nabla^{2}\mu^{\left(i\right)}\left(r\text{,}t\right)=\sum_{k}v_{k}^{\left(i\right)}\left(t\right)\nabla^{2}w_{k}^{\left(i\right)}\left(r\right)=\sum_{k}\lambda_{k}^{\left(i\right)}v_{k}^{\left(i\right)}\left(t\right)w_{k}^{\left(i\right)}\left(r\right)\text{.} \end{array}$$

The input *f*^(i)^(**r**,*t*) and its stimulus-related component can also be expressed as a transform pair

(A12) $$\begin{array}{c} f^{\left(i\right)}\left(r\text{,}t\right)=\sum_{k}f_{k}^{\left(i\right)}\left(t\right)w_{k}^{\left(i\right)}\left(r\right)\\ f_{k}^{\left(i\right)}\left(t\right)=\int w_{k}^{\left(i\right)}\left(r\right)f^{\left(i\right)}\left(r\text{,}t\right)dr\\ u\left(r\text{,}t\right)=\sum_{k}u_{k}\left(t\right)w_{k}^{\left(i\right)}\left(r\right)\\ u_{k}\left(t\right)=\int w_{k}^{\left(i\right)}\left(r\right)u\left(r\text{,}t\right)dr. \end{array}$$

Substituting these expressions into (A10), we can use the orthogonality of the modes (A8) to eliminate terms that depend on *r* and express the temporal dynamics as an ODE

(A13) $$\begin{array}{c} {\dot{v}}_{k}^{\left(i\right)}\left(t\right)=\frac{1}{2\kappa }\left(-\sum_{k\prime }\delta_{kk\prime }{\ddot{v}}_{k\prime }^{\left(i\right)}\left(t\right)+\frac{3}{2}c^{2}\sum_{k\prime }\delta_{kk\prime }\lambda_{k\prime }^{\left(i\right)}v_{k\prime }^{\left(i\right)}\left(t\right)+\sum_{k\prime }\delta_{kk\prime }f_{k\prime }^{\left(i\right)}\left(t\right)\right)\\ =\frac{1}{2\kappa }\left(-{\ddot{v}}_{k}^{\left(i\right)}\left(t\right)+\frac{3}{2}c^{2}\lambda v_{k}^{\left(i\right)}\left(t\right)+f_{k}^{\left(i\right)}\left(t\right)\right) \end{array}$$

Rearranging (A13) and substituting for *f*_*k*_^(i)^(*t*) from (A5) and (A12) gives

(A14) $$\frac{\partial^{2}}{\partial t^{2}}v_{k}^{\left(i\right)}\left(t\right)+2\kappa \frac{\partial }{\partial t}v_{k}^{\left(i\right)}\left(t\right)+\left(\kappa^{2}-\frac{3}{2}\lambda_{k}^{\left(i\right)}c^{2}\right)v_{k}^{\left(i\right)}\left(t\right)=c\kappa \int w_{k}^{\left(i\right)}\left(r\right)\varsigma^{\left(i\right)}\left(r\text{,}t\right)dr\text{.}$$

Recall that *ρ* is the parameter of the sigmoid activation function *S*(*μ*^(*i*)^) in Eq. 3. We can further simplify the expression for the input using the first-order approximation *S*(*μ*^(*i*)^) ≈ *ρμ*^(*i*)^(*r*, *t*) to give:

(A15) $$\begin{array}{c} \varsigma^{\left(i\right)}\left(r\text{,}t\right)=\gamma_{iu}u\left(r\text{,}t\right)+\sum_{j}\gamma_{ij}S\left(\mu^{\left(j\right)}\left(r\text{,}t\right)\right)\\ u\left(r\text{,}t\right)=\sum_{k}u_{k}\left(t\right)w_{k}^{\left(i\right)}\left(r\right)\\ S\left(\mu^{\left(i\right)}\right)=\rho \mu^{\left(j\right)}\left(r\text{,}t\right)=\rho \sum_{k}v_{k}^{\left(j\right)}\left(t\right)w_{k}^{\left(j\right)}\left(r\right)\text{.} \end{array}$$

Here, we have made the simplifying assumption that all the populations have the same spatial support and modes; and that the coupling between layers, γ_*ij*_ is uniform. This allows us to further approximate the input for the *k*-th mode with:

(A16) $$\begin{array}{c} \int w_{k}^{\left(i\right)}\left(r\right)\varsigma^{\left(i\right)}\left(r\text{,}t\right)dr\approx \gamma_{iu}\sum_{k\prime }\delta_{kk\prime }u_{k\prime }\left(t\right)+\sum_{j}\gamma_{ij}\rho \sum_{k\prime }\delta_{kk\prime }v_{k\prime }^{\left(j\right)}\left(t\right)\\ \approx \gamma_{iu}u_{k}\left(t\right)+\sum_{j}\gamma_{ij}S\left(v_{k}^{\left(j\right)}\left(t\right)\right) \end{array}$$

In effect, the modes are uncoupled by their orthogonality. This means the mean-field effects are only communicated within, not between modes. Substituting (A16) into (A14) gives an approximate ODE for the temporal expression of each mode:

(A17) $$\begin{array}{c} {\ddot{v}}_{k}^{\left(i\right)}\left(t\right)+2\kappa {\dot{v}}_{k}^{\left(i\right)}\left(t\right)+\left(\kappa^{2}-\frac{3}{2}\lambda_{k}^{\left(i\right)}c^{2}\right)v_{k}^{\left(i\right)}\left(t\right)=c\kappa \varsigma_{k}^{\left(i\right)}\left(t\right)\\ \varsigma_{k}^{\left(i\right)}\left(t\right)=\gamma_{iu}u_{k}\left(t\right)+\sum_{j}\gamma_{ij}S\left(v_{k}^{\left(j\right)}\left(t\right)\right)\text{.} \end{array}$$

If we retain only the fundamental mode, then *λ*_*k*_^(*i*)^ = *λ*_0_^(*i*)^ = 0 and (A17) has exactly the same form as the neural-mass model in Eq. 3 but where the mean depolarisation is replaced by the eigenfunction of each mode. This also describes the fluctuations of the standing-wave in Eq. 9.

More generally, when different populations have different spatial modes (i.e., populations in different cortical regions), one would have to replace *γ*_*ij*_ with Γ_*kk*__′_^(*ij*)^ and sum over modes and populations; where the parameter Γ_*kk*__′_^(*ij*)^ couples the *k*-th mode of the *i*-th population to the *k*′-th mode in population *j*. This parameter can model inhomogeneous extrinsic connections that couple spatial modes in different parts of the brain; this is the implicit meaning of *γ*_*ij*_ = Γ_00_^(*ij*)^ in the main text.

## Footnotes

1 When considering 2D neural fields on the cortical manifold, Eq. 3 is an approximation that is valid whenever the spatial decay of lateral interaction is short enough (typically smaller than the average distance between two cortical sulci; see Appendix 1).
